# Detailed Clinical, Ophthalmic, and Genetic Characterization of *ADGRV1*-Associated Usher Syndrome

**DOI:** 10.1016/j.ajo.2023.06.026

**Published:** 2023-12

**Authors:** Malena Daich Varela, Shiao Wei Wong, Gulunay Kiray, Patricio G. Schlottmann, Gavin Arno, Amjaad N. Abu Shams, Omar A. Mahroo, Andrew R. Webster, Alaa AlTalbishi, Michel Michaelides

**Affiliations:** a From the Moorfields Eye Hospital (M.D.V., S.W.W., G.K., G.A., O.A.M., A.R.W., M.M.), London, UK; b UCL Institute of Ophthalmology, University College London (M.D.V., G.A., O.A.M., A.R.W., M.M.), London, UK; c Organizacion Medica de Investigacion, Buenos Aires, Argentina (P.G.S.); d St John of Jerusalem Eye Hospital Group, Jerusalem, Palestine (A.N.A.S., A.A.T.)

## Abstract

**Purpose:**

To present the clinical characteristics, retinal features, natural history, and genetics of *ADGRV1*-Usher syndrome (USH).

**Design:**

Multicenter international retrospective cohort study.

**Methods:**

Clinical notes, hearing loss history, multimodal retinal imaging, and molecular diagnosis were reviewed. Thirty patients (28 families) with USH type 2 and disease-causing variants in *ADGRV1* were identified. Visual function, retinal imaging, and genetics were evaluated and correlated, with retinal features also compared with those of the commonest cause of USH type 2, *USH2A*-USH.

**Results:**

The mean age at the first visit was 38.6 ± 12.0 years (range: 19-74 years), and the mean follow-up time was 9.0 ± 7.7 years. Hearing loss was reported in the first decade of life by all patients, 3 (10%) described progressive loss, and 93% had moderate-severe impairment. Visual symptom onset was at 17.0 ± 7.7 years of age (range: 6-32 years), with 13 patients noticing problems before the age of 16. At baseline, 90% of patients had no or mild visual impairment. The most frequent retinal features were a hyperautofluorescent ring at the posterior pole (70%), perimacular patches of decreased autofluorescence (59%), and mild-moderate peripheral bone-spicule–like deposits (63%). Twenty-six (53%) variants were previously unreported, 19 families (68%) had double-null genotypes, and 9 were not-double-null. Longitudinal analysis showed significant differences between baseline and follow-up central macular thickness (−1.25 µm/y), outer nuclear layer thickness (−1.19 µm/y), and ellipsoid zone width (−40.9 µm/y). The rate of visual acuity decline was 0.02 LogMAR (1 letter)/y, and the rate of constriction of the hyperautofluorescent ring was 0.23 mm^2^/y.

**Conclusions:**

*ADGRV1*-USH is characterized by early-onset, usually non-progressive, mild-to-severe hearing loss and generally good central vision until late adulthood. Perimacular atrophic patches and relatively retained ellipsoid zone and central macular thickness in later adulthood are more often seen in *ADGRV1-*USH than in *USH2A*-USH.

Concurrent visual and hearing loss (also known as deaf blindness) affects approximately 1 in 10,000 individuals.^1^ It can occur secondary to infections (such as rubella and cytomegalovirus), perinatal complications, or genetic abnormalities.^2^ Among the latter, the most common is Usher syndrome (USH), and rarer etiologies include peroxisomal disorders, CHARGE, Marshall, or Wolfram syndrome.^2^, ^3^, ^4^, ^5^ USH may be classified clinically depending on the severity of deafness, the coexistence of vestibular dysfunction, and the disease onset.^6^

Broadly, USH type 1 is the most severe, with profound, congenital sensorineural hearing loss, vestibular dysfunction (resulting in balance issues), and childhood-onset retinitis pigmentosa (RP).^7^ It accounts for around one-third of all cases of USH and is caused by disease-causing variants primarily in *MYO7A, CDH23, USH1C, PCDH15*, or *USH1G*.^8^^,^^9^ USH type 2 typically presents with moderate sensorineural hearing loss since childhood and RP diagnosed in adolescence.^9^ It is the most common subtype, accounting for nearly two-thirds of cases, and is primarily due to biallelic variants in *USH2A, ADGRV1*, or *WHRN*.^10^ USH types 3 and 4 are rare and seen in specific populations (eg, Finnish and Ashkenazi Jewish); they are characterized by childhood-onset hearing loss and adult-onset RP, secondary to pathogenic variants in *CLRN1* and *ARSG*, among others.^6^^,^^7^^,^^11^

Approximately 90% of patients with USH type 2 (USH2) have biallelic pathogenic variants in *USH2A* (USH2A), 9% in *ADGRV1* (USH2C), and 1% in *WHRN* (USH2D)*.*^12^ USH2B has been mapped to 3p23-p24.2; however, the gene has not been identified.^13^ *USH2A* is also the most common gene to cause nonsyndromic autosomal recessive RP; thus there is substantial information about its disease mechanism and associated phenotypes.^9^^,^^14^, ^15^, ^16^ In stark contrast, there are limited data about *ADGRV1*-associated USH in the literature.

*ADGRV1* (MIM *602851, also known as *GPR98, MASS1,* and *VLGR1*) is associated with 5% of all visually and hearing impaired patients and is the fourth most common gene in USH (after *USH2A, MYO7A*, and *CDH23*).^12^ It is located on chromosome 5q14.3, spans 90 exons, 6306 amino acids, and encodes the largest G protein–coupled receptor-1, ubiquitously expressed throughout the body.^17^^,^^18^ ADGRV1 belongs to the Usherin protein network, interacting with other USH proteins and functioning as a fibrous membrane linkage in cilia of inner-ear hair cells and photoreceptors.^11^^,^^19^ It is a transmembrane protein with an extracellular portion of 35 sodium-calcium exchangers (Calx-beta domains) and a block of epilepsy-associated repeat domains.^20^ It is also highly expressed in the developing central nervous system and has been the focus of recent research given its association with epilepsy.^17^^,^^21^ Herein, we describe the detailed clinical and genetic characteristics of the largest cohort of patients with *ADGRV1*-associated USH to date.

## METHODS

This study is a retrospective consecutive case series of patients who attended Moorfields Eye Hospital (London, UK), St. John of Jerusalem Eye Hospital (Jerusalem, Palestine), and Organizacion Medica de Investigacion (Buenos Aires, Argentina) with USH, and who were found to have biallelic rare or likely disease-causing variants in *ADGRV1*. Patients from Moorfields Eye Hospital were identified through the inherited eye disease database. Informed consent was obtained from all patients. Ethical approval was provided by the local ethics committee, and the study honored the tenets of the Declaration of Helsinki.

Relevant patient data were retrieved from the electronic health care records and imaging software systems. The age of disease onset was defined as the age of the first disease related symptom(s). Snellen visual acuities were recorded and converted to logMAR for descriptive statistics. Count fingers vision was given a value of logMAR 1.98, hand motion logMAR 2.28, light perception logMAR 2.7, and no light perception logMAR 3.0.^22^^,^^23^ Asymmetric best corrected visual acuity (BCVA) was defined as a difference of ≥0.3 logMAR (equivalent to 15 Early Treatment Diabetic Retinopathy Study letters) between eyes. Patients were categorized using the World Health Organization visual impairment criteria, which define no or mild visual impairment as BCVA ≤0.48 (6/18, 20/60), moderate impairment as BCVA >0.48 and ≤1.0 (6/60, 20/200), severe as BCVA >1.0 and ≤1.3 (3/60, 20/400), and blindness as BCVA >1.3. Records of visual field were very limited within our cohort; therefore, we only took into consideration BCVA to classify patients.

Further clinical assessments consisted of dilated fundus examination, spectral-domain optical coherence tomography (OCT, Heidelberg Spectralis; Heidelberg Engineering, Inc, Heidelberg, Germany), fundus autofluorescence (Heidelberg Spectralis and Optos PLC, Dunfermline, UK), and ultrawide field fundus color photography (Optos PLC). OCT thickness in the general population was extracted from Invernizzi and associates.^24^ Ellipsoid zone (EZ) width was measured at the foveal scan, photoreceptor outer segment length (PROS) was obtained by measuring the distance between the inner border of the EZ and the inner border of the retinal pigment epithelium (RPE), and foveal outer segment pigment epithelial thickness (FOSPET) was derived from calculating the distance between the EZ and the outer border of the RPE.^25^ The FOSPET-PROS ratio (FPR) was determined by dividing FOSPET by PROS.

DNA was extracted from whole blood, and genetic testing was performed using panel-based targeted next-generation sequencing, whole exome sequencing, or whole genome sequencing. Where appropriate and available, blood samples were taken from parents or siblings to confirm the segregation of proposed variants. In silico analysis was performed for previously unreported variants. The pathogenicity of each variant was classified according to the guidelines of the American College of Medical Genetics and Genomics.^26^, ^27^, ^28^ Disease-specific guidelines to apply PP4 were extracted from Oza and associates.^29^

GraphPad Prism 8.0.2 (GraphPad Software) was implemented for statistical analysis. The threshold of significance was set at *P* < .05. Linear regressions and *t*-test were used for parametric variables’ assessment, Welch's *t*-test variation was used when the sample sizes were significantly different. Kaplan-Meier survival curves were used to determine the percentage of patients with a certain level of visual acuity (VA) at different decades of life.

## RESULTS

### Demographics, Phenotype, and Visual Acuity

Thirty patients from 28 families were identified. Their clinical characteristics are listed in Table 1 and Supplemental Table 1. Eleven (37%) individuals were female and 19 (63%) were male. The age at their first visit was 38.6 ± 12.0 years (mean ± SD; median: 37.5, range: 19-74). All patients had hearing impairment: 14 congenital and 16 since childhood. Three patients (10%) described progressive hearing loss. Twenty-eight (93%) patients reported moderate-severe hearing loss, with 27 using hearing aids and 1 having a cochlear implant. The remaining 2 patients had mild impairment without the need for hearing aids. No neurological issues were reported. Among the patients who reported their ethnicity (83%), 8 were Asian, 16 were White, and 1 was mixed. Consanguinity was reported in 5 families, where parents were first cousins.

**Table 1:** Clinical Characteristics of Patients With *ADGRV1-*Associated Usher Syndrome.

Characteristics	Patients (n = 30)
Families	28
Gender at birth, n (%)	
Female	11 (37)
Male	19 (63)
Age at first examination, mean ± SD (y)	38.6 ± 12
Age at last examination, mean ± SD (y)	49.9 ± 13.2
Follow-up time, mean ± SD (y)	9 ± 7.7
Ethnicity, n (%)	25 (83)
Asian	8 (27)
White European	16 (53)
Mixed	1 (3)
Age of onset visual disturbances, mean ± SD (y)	17 ± 7.7
Pediatric, n (%)	13 (43)
Adult, n (%)	17 (57)
Hearing impairment onset, n (%)	
Congenital	14 (47)
Childhood	16 (53)
Reported first symptom, n (%)	
Night blindness	21 (70)
Concurrent nyctalopia and peripheral field loss	9 (30)
Posterior pole characteristics, n (%)	
Decreased autofluorescent perimacular lesions	16 (53)
Hyperautofluorescent ring at the posterior pole	21 (70)
Peripheral retinal pigment deposits, n (%)	
None	5 (17)
Minimal/moderate	19 (63)
Dense	6 (20)

The mean age of visual symptom onset was 17.0 ± 7.7 years (median: 15.0, range: 6-32), with 13 patients symptomatic before 16 years of age (43%). Twenty-one (70%) patients described nyctalopia as their first symptom, and 9 (30%) reported concurrent poor night vision and peripheral field loss. At their initial visit, 2 patients had moderate visual loss (39 and 61 years old), 1 was blind (40 years old), and the rest had none or mild visual impairment (90%, 19-74 years old).

At baseline, BCVA was 0.3 ± 0.4 logMAR OD and OS. Asymmetric BCVA was seen in 4 (13%) patients. Analyzed cross-sectionally, there was no significant association between age and BCVA OD (*P* = .07) or OS (*P* = .25).

### Clinical Examination, Color and Autofluorescence Fundus Imaging

Twenty (67%) individuals had cataracts or were pseudophakic in at least 1 eye at baseline. Nineteen (63%) patients had symmetric mild-moderate pigmented bone-spicule–like (BSL) deposits (19-74 years old), 6 (20%) had severe, dense pigmentary change (36-50 years old), and 5 (17%) had no pigment deposits (25-35 years old).

**Figure 1:**
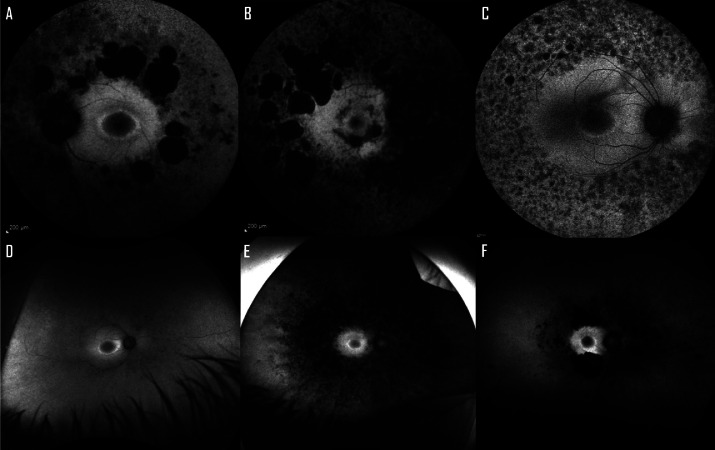
Autofluorescence images of patients with *ADGRV1*-Usher syndrome. A. A 68-year-old patient with a hyperautofluorescent perimacular ring (HPR) and patches of definitely decreased autofluorescence (DDAF) at the level of the vascular arcades. B. A 44-year-old patient with parafoveal atrophy and DDAF in the mid-periphery. C. A 33-year-old patient with an HPR, mid-peripheral DDAF, and granular hypoautofluorescence. D. A 30-year-old patient with an HPR and largely preserved peripheral autofluorescence, with mild granular hypoautofluorescent changes in the mid-periphery. E. A 54-year-old patient with a HPR and severe mid-peripheral granular and patch-like atrophy, predominantly nasal. F. A 69-year-old patient with a maintained HPR, DDAF patches over the arcades, and granular hypoautofluorescence mainly in the nasal retina.

Twenty-one patients had bilateral hyperautofluorescent rings at the posterior pole, 3 had frank parafoveal atrophy (44-74 years old), and 4 had normal macular autofluorescence (49-50 years old).^30^ The initial area of the hyperautofluorescent ring was 7.42 ± 8.36 mm^2^ OD and 7.58 ± 8.86 mm^2^ OS, and it was not significantly associated with age (*P* = .22) or BCVA (*P* = .06 and .25). Perimacular patches of decreased autofluorescence were seen around the vascular arcades bilaterally in 16 patients, from as early as 26 years of age (53%, Figure 1, A-C), and unilaterally in 2 (7%). Ultrawide field fundus autofluorescence imaging was available in 23 (77%) patients. The retinal periphery was characterized by patchy and granular hypoautofluorescent lesions in 9 patients (30%, denser in the mid-periphery, Figure 1, D-F), hypoautofluorescence with BSL pigment in 6 (20%), granular pattern in 4 (13%), dispersed hypoautofluorescent patches in 2 (7%), normal in 1 (3%), and had bilateral preserved para-arteriolar RPE changes in the last patient (3%).

### Macular OCT Analysis

Twenty-seven (90%) patients had macular OCT. Twenty-one (70%) patients had bilateral epiretinal membranes, and one had unilateral epiretinal membrane. Two (7%) patients had a unilateral macular hole, 11 (37%) had bilateral cystoid macular edema (CMO), and 4 (13%) had unilateral CMO; hence, they were excluded from the quantitative thickness analysis (n = 26 eyes included). The age of patients with CMO was 41.8 ± 12.7 years vs the age of those without CMO, 36.6 ± 10.7 years (*P* = .25).

**Figure 2:**
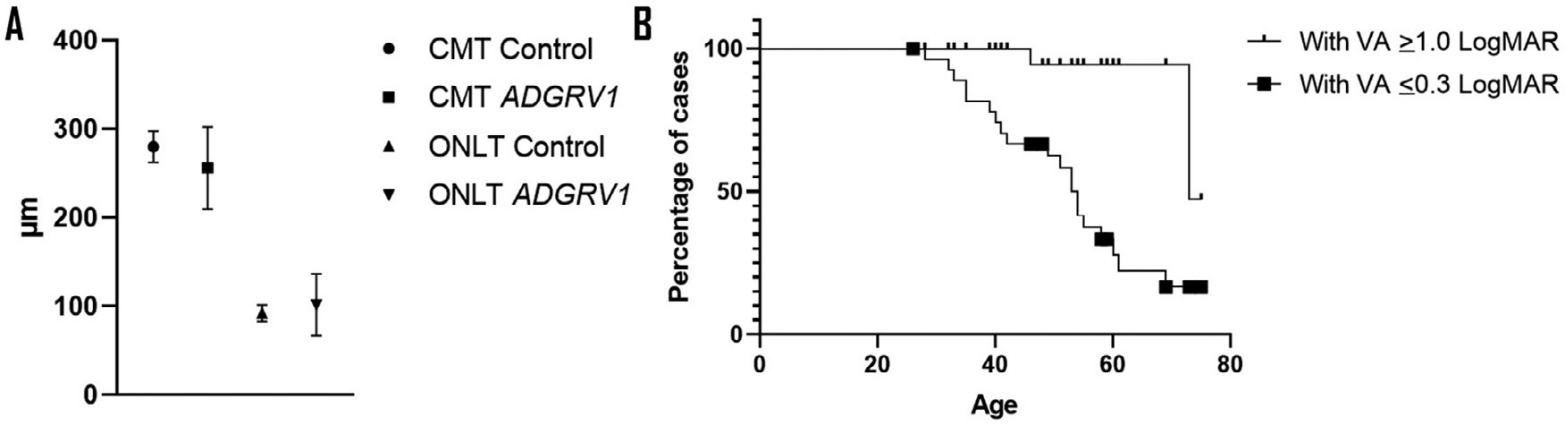
Optical coherence tomography in *ADGRV1*-Usher syndrome. A. Central macular thickness (CMT) in our cohort was significantly lower compared with the unaffected population (*P* = .01), and outer nuclear layer thickness (ONLT) was similar (*P* = .15). B. Kaplan-Meier survival analysis showing the percentage of patients with BCVA ≤0.3 logMAR (6/12) in at least 1 eye (74% of the patients at 40 years of age and 50% at 53 years of age) and with legal blindness (≥1 logMAR, 50% at 73 years of age). BCVA = best corrected visual acuity.

Baseline central macular thickness (CMT) was 255.4 ± 44.0 µm OD and 257.6 ± 46.4 µm OS. Outer nuclear layer thickness (ONLT) was quantifiable in 25 eyes (19-74 years old), with a mean value of 95.9 ± 31.4 µm OD and 101.6 ± 34.8 µm OS. CMT was significantly lower than in unaffected population (*P* = .01), whereas no significant differences were found in ONLT (*P* = .15, Figure 2, A). EZ was present in 26 patients at baseline (50 eyes, 19-74 years old) with a mean width of 2296 ± 1209 µm OD and 2324 ± 1330 µm OS. FOSPET was 64.7 ± 17.0 µm OD and 61.8 ± 21.6 µm OS, and PROS was 42.1 ± 12.0 µm OD and 43.6 ± 11.6 µm OS; both decreased compared with unaffected population.^31^^,^^32^ The mean FPR (FOSPET-PROS ratio [FPR]) was 1.58 ± 0.3 OD and 1.49 ± 0.2 OS. There were no significant differences between parameters in OD vs OS, indicating high interocular symmetry (*P* = .92-.96).

Considering cross-sectional data only, a significant association was observed between older age and decreased PROS (0.0001 OD and 0.03 OS), FOSPET (0.03 OD and 0.0004 OS), and FPR OD (0.0009). No significant association was found between age, CMT, ONLT, EZ width, and FPR OS (*P* = .17-.71). BCVA was significantly associated with CMT OD (*P* = .04) and EZ width OS (*P* = .04), and not with CMT OS, EZ OD, ONLT, FOSPET, PROS, or FPR (*P* = .15-.89). There were no significant differences regarding the BCVA of patients with and without CMO (*P* = .9).

### Longitudinal Analysis

The mean follow-up time was 9 ± 7.7 years (0-24 years), and the mean age at the final visit was 49.9 ± 13.2 years. Final BCVA was 0.5 ± 0.6 logMAR OD and 0.6 ± 0.7 OS. Asymmetric BCVA was seen in 5 (17%) patients. The rate of BCVA decline was 0.02 logMAR (1 letter)/year, and there was a significant difference between baseline and follow-up BCVA (*P* < .0001). Ten (33%) patients lost at least 15 Early Treatment Diabetic Retinopathy Study letters or more over follow-up in 1 or both eyes (after 3-17 years). Four (13%) patients progressed to more advanced World Health Organization categories of visual impairment over follow-up, 1 of whom became blind.

Kaplan-Meier survival analysis predicted that at 40 years of age, 74% of the patients have 0.3 logMAR (6/12) in at least 1 eye and this drops to 50% at 53 years of age. It also predicted that at 73 years of age, 50% of the patients will reach legal blindness based on VA (≥1 logMAR, Figure 2, B).

Fifteen patients still had bilateral hyperautofluorescent macular rings at the posterior pole, with a mean area of 4.6 ± 3.7 mm^2^ OD and 4.4 ± 3.3 mm^2^ OS, significantly smaller than baseline (*P* = .004 OD and .006 OS). The rate of constriction of the hyperautofluorescent ring was 0.23 mm^2^/year.

Twenty-five (83%) patients had follow-up macular OCT scans. Three eyes lost discernible EZ, and the latest mean width was 1672.1 ± 1194.1 OD and 1641.5 ± 168.8 OS µm. There were no significant differences between EZ OD vs OS (*P* = .98), with high interocular symmetry.

Longitudinal analysis demonstrated significant differences between baseline and follow-up CMT (*P* = .02, −1.25 µm/y), ONLT OS (*P* = .01, −1.19 µm/y), EZ width (*P* = .001, −40.9 µm/y), and PROS (*P* = .03, −0.36 µm/y).

BCVA OS was significantly associated with CMT (*P* = .015), PROS (*P* = .003), and FOSPET (*P* = .004). BCVA OD was not associated with any structural parameter (*P* = .09-.87).

### Molecular Genetics

**Figure 3:**
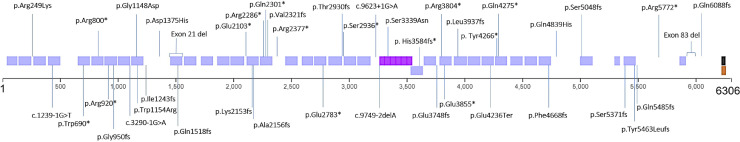
Graphical representation of ADGRV1 protein and the variants in this cohort (www.uniprot.org). The light purple functional domains correspond to the sodium-calcium exchangers (Calx-beta domains), those in dark purple represent the epilepsy-associated repeats (EAR), and the last ones represent the transmembrane region. Variants displayed above the protein are the ones already reported in the literature, and those below are the previously unreported novel variants described in this study.

All patients had biallelic variants in *ADGRV1* and 7 were homozygous. A total of 49 different variants were present in our cohort: 32 nonsense, 10 missense, 4 splice-site alterations, 2 large deletions, and 1 deep-intronic variant. Twenty-three (47%) were previously reported,^33^, ^34^, ^35^, ^36^, ^37^, ^38^, ^39^, ^40^, ^41^ and 26 (53%) were novel variants (Supplemental Table 2). Of these, 5 were classified as pathogenic, 16 as likely pathogenic, and 5 as variants of uncertain significance. A schematic representation of pathogenic and likely pathogenic variants found in our cohort is presented in Figure 3.

Only 2 variants were found in more than 1 family: c.3443G>A p.(Gly1148Asp) and c.6901C>T p.(Gln2301*), present in families of White British origin. The remaining 47 variants were seen once in 1 family of the cohort.

Nineteen (68%) families had double-null genotypes (DN; nonsense, frameshift, splicing, or exon deletion), and 9 were not-double-null (NDN); 7 (25%) compound heterozygous of a null and a missense variant, and 2 (7%) had 2 missense changes. Three patients (ID 4, 11, and 27) had USH2, harbored a pathogenic variant and a variant of uncertain significance, had other known USH genes excluded, and hence were included in the cohort and considered likely *ADGRV1*-related cases.

### Genotype-Phenotype Correlation Analysis

Patients with a DN genotype were younger than NDN patients at baseline, 35.2 ± 10.1 years versus 45.4 ± 13.6 years (*P* = .07), and had earlier age of onset, 15.4 ± 7.3 versus 18.6 ± 9.2 (*P* = .48). No relevant differences were seen in degree and onset of hearing loss.

Regarding retinal features, 4 of the 6 patients with severe BSL were NDN, and 8 of 9 patients without perimacular atrophic patches were DN. No significant differences were found in structural parameters such as hyperautofluorescent ring area (*P* = .6), EZ width (*P* = .23), CMT (*P* = .12), and ONLT (*P* = .06).

Baseline BCVA of DN and NDN groups were not significantly different (*P* = .34), with the caveat that the NDN group was a decade older. Analyzed at a similar age, patients with DN had significantly worse VA (0.7 ± 0.82) compared with NDN (0.26 ± 0.18, *P* = .04). The only blind patient at baseline and one of those with moderate visual impairment were DN, and 3 of the 4 who progressed to more advanced stages were DN. The rate of VA decline was the same in both DN and NDN groups.

## DISCUSSION

This study describes the phenotype of the largest cohort of patients with *ADGRV1*-associated USH to date. Hearing loss, retinal features, and longitudinal evaluation are detailed, establishing useful diagnostic features and describing the severity of disease progression, aiming at informing patients’ prognosis and management. Twenty-six previously unreported variants are included, and genotype-phenotype correlations are drawn.

USH2 is characterized as having moderate-severe hearing impairment, with onset generally in the first decade of life and up to the early 30s.^42^, ^43^, ^44^ A progressive deterioration has been reported in *USH2A*-USH2, with mild annual deterioration.^45^ *ADGRV1* has been previously characterized as having more severe and slightly later onset hearing loss than *USH2A*.^43^^,^^46^ Postlingual hearing loss has even been described in 1 family.^47^ In our cohort, all patients reported hearing impairment in the first decade of life, only 10% reported having progressive hearing issues, and 2 of them had mild impairment (both DN). Although longitudinal audiological assessment would shed light on the characteristics of the hearing loss, it appears that *ADGRV1*-USH2 can present with a rather stable hearing loss, with onset generally before 10 years of age, and in some cases, relatively mild.

Individuals with *ADGRV1*-USH have been described as possibly having older age of visual symptom onset than patients with *USH2A*.^35^ However, the patients presented herein with *ADGRV1*-USH presented with visual symptoms in the second/early third decade of life, as classically described in USH2.^7^ Therefore, the age of onset in our cohort was similar to that seen in patients with *USH2A*-USH (adolescence/early adulthood).^16^^,^^44^^,^^48^^,^^49^

Similar to *USH2A*, baseline BCVA was ≤0.4 logMAR, and a minority of patients (10%) had moderate or worse visual impairment.^16^^,^^35^^,^^48^^,^^50^ As previously noted for both *USH2A* and *ADGRV1*, BCVA was significantly associated with age.^49^, ^50^, ^51^ The rate of BCVA decline appeared both slower (1 vs approximately 2 letters/y)^48^ and faster than in *USH2A* (5% vs 2.5%/y), depending on the study of comparison.^52^ Central vision was largely maintained, with patients in their fifth decade still having 0.0 logMAR vision and 74% of patients having 0.3 logMAR (6/12) until 40 years of age. Fakin and associates^49^ reported a 50% chance of reaching legal blindness based on VA at 64 years of age, but we found this to be at 73 years of age, similar to the value reported for *USH2A*-USH (74 years old).^53^

**Figure 4:**
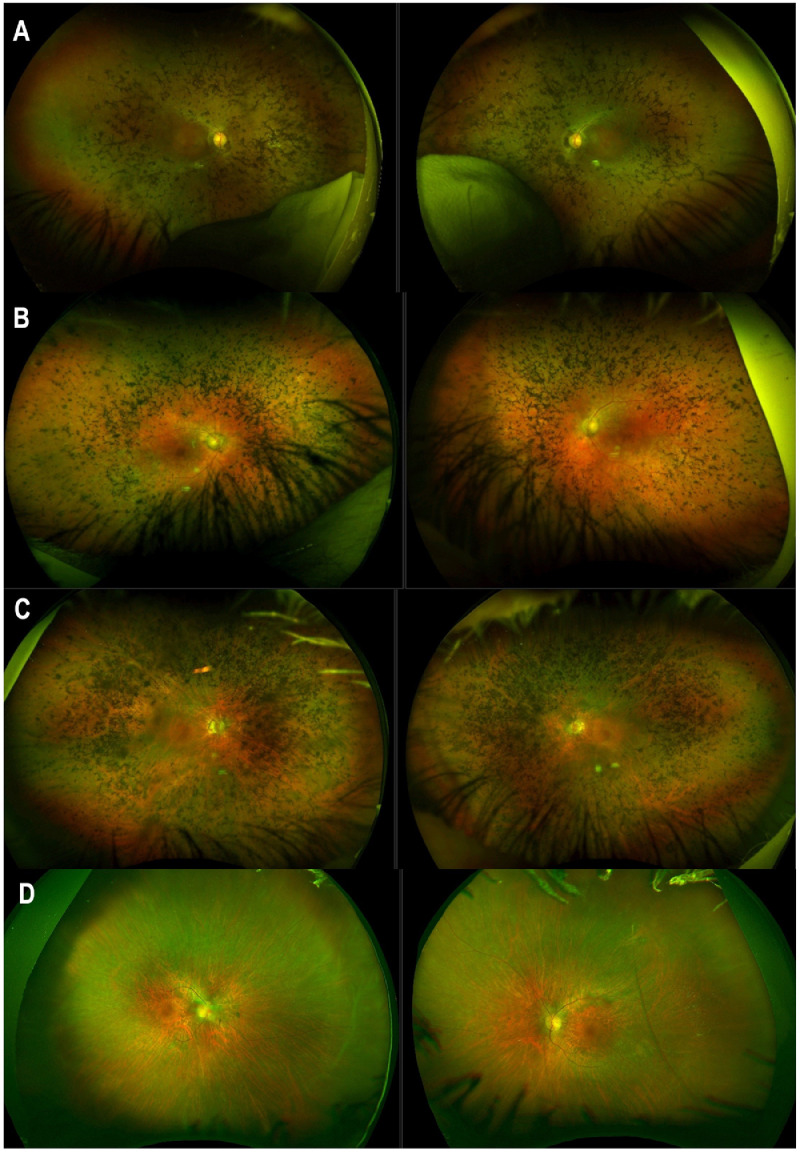
Ultrawide color fundus features of patients with Usher syndrome (USH). A. A 53-year-old patient with *USH2A*-USH, with moderate bone-spicule–like (BSL) pigment clumps and pink optic discs. B. A 60-year-old patient with *MYO7A*-USH, with moderate BSL pigment throughout the retina and vessel thinning. C. A 56-year-old patient with *ADGRV1*-USH, with severe, deep BSL and nummular pigment affecting the whole retina. D. A 49-year-old patient with *ADGRV1*-USH, with vessel thinning and no retinal pigmented deposits. (For interpretation of the references to colour in this figure legend, the reader is referred to the web version of this article.)

Cataracts affected 67% of the patients at baseline, which was a similar percentage to *USH2A* (79%) and *MYO7A* (52.5%-60%).^48^^,^^54^ Regarding retinal features, the majority of patients (80%) had no to moderate pigmentary changes, which is also in keeping with *USH2A*- and *MYO7A-*USH (Figure 4).^54^, ^55^, ^56^ The remaining few patients had dense pigmentary changes primarily in the mid-periphery, a phenotype not previously described in patients with USH2.

A hyperautofluorescent ring at the posterior pole was the most common feature in our patients, and this has previously been described both for *USH2A* and *ADGRV1*.^49^ Unlike previous reports, we did not find a strong association between age and ring area, with patients in the sixth and seventh decade of life still having a preserved hyperautofluorescent ring.^49^ The rate of constriction was similar to that reported in *USH2A* (3.8% vs 3.5%).^52^ Perimacular atrophic patches have been described in patients with both *ADGRV1*- and *USH2A*-USH2, in a minority of patients, from 31 years of age.^49^ In our cohort, these affected more than half of the patients, from 26 years of age (Figure 1, A-C), apparently more common and of earlier onset than in *USH2A*.

There is variability in previously reported OCT structural parameters in *USH2A*-USH; however, EZ and CMT appeared more preserved in patients with *ADGRV1* of similar age (CMT 253.3 µm vs 210.28-247 µm, EZ 2296 µm vs 1307-2155 µm).^48^^,^^50^^,^^57^, ^58^, ^59^ In a previous report of 3 sisters, ONLT appeared reduced; however, in our larger cohort, we found it similar to unaffected population.^51^

*ADGRV1-*USH shows broad allelic heterogeneity. Eighty percent of the variants in our cohort were null, matching the previously described, possibly high tolerance of *ADGRV1* to missense variants.^46^ The large majority of the variants were present only in 1 family of the cohort. This is perhaps due to a loss-of-function mechanism in a large gene with a low incidence, which leads to a diverse range of variants distributed along the entire length of the gene (Figure 3). Of the previously reported variants, 24 were seen in USH2 only, whereas the 2 missense variants p.Ile2332Phe and p.Gly1148Asp were also associated with nonsyndromic hearing loss and p.Gly1148Asp with isolated retinal degeneration.^39^^,^^60^, ^61^, ^62^

It has previously been described that missense variants in *ADGRV1* can be associated with isolated hearing loss,^63^ with currently 55% of reported missense variants associated with isolated hearing loss, vs 45% with USH2 (https://my.qiagendigitalinsights.com/bbp/view/hgmd/.php, reviewed in March 2023), without clear clusters or hot spots. Hence, we can infer that patients carrying at least 1 missense variant can still present as USH2. Interestingly, some functional features (onset of visual symptoms and BCVA) were more severe in patients with DN genotype, whereas retinal degeneration was more visible in NDN. This dissociation could be related to the limited number of patients in our cohort or could represent a possible toxic effect on the retina by mutant alleles.^64^

Our study's strengths are the relatively large number of genetically confirmed patients, their different ethnic background, and the inclusion of centers from different countries. Some of its limitations are the retrospective nature of the study, the larger proportion of individuals with DN genotype vs NDN, incomplete data for some individuals, and nonstandardized methods and protocols throughout participating centers.

*ADGRV1* is a very large gene, greatly exceeding the adeno-associated virus packaging capacity, thereby requiring an alternative approach or multiple adeno-associated virus vector-based delivery for gene supplementation.^65^ Gene independent strategies reducing cell death through neuroprotection and antioxidants may be a suitable alternative for these patients.^66^ An ongoing phase I/II clinical trial testing an oral antioxidant in patients with USH may provide useful information regarding this option (NCT04355689).

In conclusion, this paper describes the largest cohort of patients with *ADGRV1*-USH to date. Multimodal clinical features, disease and rates of progression, and genotype-phenotype correlations are detailed. Patients with *ADGRV1*-USH have hearing loss early in life, which is usually non-progressive, and may be mild to severe. They usually maintain good central vision until late adulthood, with relatively preserved structural and functional parameters. Fundus evaluation commonly depicts a mild-to-moderate pigmentary degeneration, although more severe cases can also occur. Compared with the more prevalent *USH2A*-USH, the patients in the present study had a similar age of visual symptom onset, baseline BCVA, and rate of BCVA deterioration; structurally, perimacular atrophic patches were seen more often in *ADGRV1*, and EZ and CMT appeared relatively more preserved.

## Appendix Supplementary materials

Image, application 1

Image, application 2
