# Supplementary material for: Detailed Clinical, Ophthalmic, and Genetic Characterization of ADGRV1-Associated Usher Syndrome
Source: Am J Ophthalmol. 2023 Dec;256:186–95. doi: 10.1016/j.ajo.2023.06.026 (PMC11139646; doi:10.1016/j.ajo.2023.06.026)
Supplement: Supplementary file 1 [file mmc1.docx]

Supplemental Material

Supplemental Table 1. Clinical, Genetic, and Imaging Characteristics of the ADGRV1-Usher Syndrome Cohort

| ID | GC | Gender | Ethnicity | Age at First Visit | Age of Onset of Hearing Loss | Degree of Hearing Loss | Progressive Hearing Loss | Age of Onset Vision Loss | Retinal Pigment | Macular AF Pattern | Perimacular hypo-AF Patches? | Presenting BCVA (logMAR) | | Genotype | |
| --- | --- | --- | --- | --- | --- | --- | --- | --- | --- | --- | --- | --- | --- | --- | --- |
|  |  |  |  |  |  |  |  |  |  |  |  | OD | OS | Allele 1 | Allele 2 |
| 1 | 43080 | F | Asian Indian | 21 | Congenital | Moderate/severe | No | 15 | No pigment | Macular ring | No | 0 | 0.18 | c.4553_4554del, p.(Gln1518fs*) | c.4553_4554del, p.(Gln1518fs*) |
| 2 | 41904 | F | Asian | 25 | 7 years old | Moderate/severe | No | NA | Mild BSL pigment | Macular ring | No | 0.18 | 0.18 | c.11410C>T, p.(Arg3804*) | c.11410C>T, p.(Arg3804*) |
| 3 | ARG | F | White | 25 | 6 years old | Moderate/severe | No | 15 | No pigment | Macular ring | No | 0 | 0 | c.11563G>T, p.(Glu3855*) | c.18261del, p.(Gln6088Serfs*20) |
| 4 | 41425 | M | NA | 26 | Congenital | Moderate/severe | No | 10 | Moderate BSL pigment | Macular ring | Bilateral | 0.18 | 0.48 | c.2239A>G, p.(Arg747Gly) | c.16453_16454del, p.(Gln5485Aspfs*2) |
| 5 | 35498 | M | White | 28 | 3 months old | Mild | No | 7 | No pigment | Macular ring | No | 0.18 | 0.18 | c.7129C>T, p.(Arg2377Ter) | c.9623+1G>A |
| 6 | PAL | M | Asian | 28 | 4 years old | Moderate/severe | Yes | 26 | Moderate BSL pigment | Macular ring | Bilateral | 0 | 0 | c.14004_14007delCTTT, p.(Phe4668Leufs*18) | c.14004_14007delCTTT, p.(Phe4668Leufs*18) |
| 7A | 23671 | M | Asian | 29 | Congenital | Moderate/severe | No | 22 | Moderate BSL pigment | Macular ring | Unilateral | 0.2 | 0.2 | Deletion of exon 83 | Deletion of exon 83 |
| 7B | 23454 | M | Asian | 30 | Congenital | Moderate/severe | No |  | Moderate BSL pigment | Macular ring | Bilateral | 0.2 | 0.3 | Deletion of exon 83 | Deletion of exon 83 |
| 8 | 9949 | F | White | 30 | Congenital | Moderate/severe | No | 22 | Moderate BSL pigment | Macular ring | Bilateral | 0 | 0 | c.11241_11242insG, p.(Val3747fs*) | c.3290-1G>A |
| 9 | 28844 | M | White | 31 | Congenital | Moderate/severe | No | 25 | Mild BSL pigment | Macular ring | No | −0.08 | −0.08 | c.14517G>C, p.(Gln4839His) | c.17314C>T, p.(Arg5772*) |
| 10 | 33857 | M | White | 33 | Congenital | Moderate/severe | No | 7 | Moderate BSL pigment | Macular ring | Bilateral | 0.18 | 0.3 | c.2070G>A, p.(Trp690*) | c.2070G>A, p.(Trp690*) |
| 11 | 21886 | F | NA | 35 | Congenital | Moderate/severe | No | 25 | No pigment | Macular ring | No | 0.14 | 0.2 | c.16111delA, p.(Ser5371fs*) | c.8156-1460A>G |
| 12 | 41060 | M | NA | 36 | Childhood | Moderate/severe | No | 26 | No pigment | Macular ring | No | 0 | −0.1 | c.6466delG, p.(Ala2l56Leufs*4) | Deletion of exon 21 |
| 13 | 9281 | M | White | 36 | Congenital | Moderate/severe | Yes | 22 | Dense BSL pigment | Macular ring | Bilateral | 0.18 | 0.18 | c.6458_6466delins8, p.(Lys2153Argfs*7) | c.4123G>C, p.(Asp1375His) |
| 14 | 31797 | F | White | 39 | 2 years old | Moderate/severe | No | 32 | Moderate BSL pigment | Macular ring | Bilateral | 0.18 | 0.18 | c.746G>A, p.(Arg249Lys) | c.6901C>T, p.(Gln2301*) |
| 15 | 22510 | F | White | 39 | Childhood | Moderate/severe | No | NA | Moderate BSL pigment | Macular ring | No | 0.18 | 1.5 | c.3726_27insA, p.(Phe1242fs*) | Deletion of exon 21 |
| 16 | 33282 | M | Asian | 39 | Early childhood | Moderate/severe | No | 6 | Mild BSL pigment | Macular ring | Bilateral | 0.6 | 0.6 | c.2758C>T, p.(Arg920*) | c.9749-2delA |
| 17 | PAL | M | Asian | 40 | Congenital | Moderate | No | 9 | Moderate BSL pigment | NA | NA | 1.98 | 1.98 | c.8347G>T, p.(Glu2783*) | c.8347G>T, p.(Glu2783*) |
| 18 | 12036 | F | White | 42 | Congenital | Severe (cochlear implant) | No | 15 | Moderate BSL pigment | Macular ring | Bilateral | 0 | 0 | c.6962_63delTG, p.(Val2321Alafs*4) | c.15144delC, p.(Ser5048Argfs*29) |
| 19 | 31392 | M | White | 42 | Childhood | Moderate/severe | No | 14 | Dense BSL pigment | Macular ring | Bilateral | 0.3 | 0.3 | c.3443G>A, p.(Gly1148Asp) | c.8807C>G, p.(Ser2936*) |
| 20 | 31198 | M | Asian Indian | 44 | Before 8 years old | Moderate/severe | No | 8 | Dense BSL pigment | Parafoveal atrophy | Bilateral | 0.18 | 0.18 | c.10873C>G, p.(Leu3625Val); c.12798T>A, p.(Tyr4266*) | c.7087G>A, p.(Glu2363Lys); c.12798T>A, p.(Tyr4266*) |
| 21 | 22577 | M | White | 47 | 5 years old | Moderate/severe | No | NA | Dense BSL pigment | Parafoveal atrophy | Bilateral | 0.2 | 0.2 | c.6856C>T, p.(Arg2286*) | c.10016G>A, p.(Ser3339Asn) |
| 22 | 38960 | F | White | 49 | 5 years old | Moderate/severe | No | 23 | Dense BSL pigment | Normal | Bilateral | 0.48 | 0.48 | c.2849del, p.(Gly950Glufs*30) | c.12823C>T, p.(Gln4275*) |
| 23 | 44244 | M | White | 50 | Congenital | Moderate/severe | No | 17 | Dense BSL pigment | Normal | Unilateral | 0.48 | 0.48 | c.8785del, p.(Thr2930Profs*12) | c.1239-1G>T |
| 24 | 45573 | M | Mixed | 50 | Childhood | Mild | No | 15 | Moderate BSL pigment | Normal | No | 0.2 | 0.2 | c.16387dup, p.(Tyr5463Leufs*8) | c.12706G>T, p.(Glu4236*) |
| 25A | 41075 | M | White | 54 | Childhood | Moderate/severe | No | NA | Moderate BSL pigment | Macular ring | Bilateral | 0.2 | 0.77 | c.3443G>A, p.(Gly1148Asp) | c.3460T>A, p.(Trp1154Arg) |
| 25B | 44165 | M | White | 74 | Congenital | Moderate/severe | No | 8 | Moderate BSL pigment | Parafoveal atrophy | Bilateral | 1 | 0.48 | c.3443G>A, p.(Gly1148Asp) | c.3460T>A, p.(Trp1154Arg) |
| 26 | 22314 | F | NA | 56 | Congenital | Moderate/severe | No | 8 | Moderate BSL pigment | NA | NA | 0.54 | 0.52 | c.6307G>T, p.(Glu2103*) | c.6901C>T, p.(Gln2301*) |
| 27 | 30233 | F | NA | 61 | Childhood | Moderate/severe | No | 30 | Moderate BSL pigment | Normal | Bilateral | 0 | 0.18 | c.2398C>T, p.(Arg800*) | c.13433G>T, p.(Ser4478Ile) |
| 28 | NA | M | White | 19 | 4 years old | Moderate/severe | No | 19 | Mild BSL pigment | Macular ring | NA | 0 | 0.1 | c.10750delC, p.(His3584Ilefs*11) | c.11808_11809insG, p.(Leu3937Alafs*32) |

AF = autofluorescence, ARG = Argentina, BCVA = best corrected visual acuity, BSL = bone spicule like, GC = XXX, NA = not applicable, PAL = Palestine.

Patients with the same ID are from the same family.
