# Supplementary material for: Detailed Clinical, Ophthalmic, and Genetic Characterization of ADGRV1-Associated Usher Syndrome
Source: Am J Ophthalmol. 2023 Dec;256:186–95. doi: 10.1016/j.ajo.2023.06.026 (PMC11139646; doi:10.1016/j.ajo.2023.06.026)
Supplement: Supplementary file 2 [file mmc2.docx]

Supplemental Table 2. Analysis of Previously Unreported *ADGRV1* Variants Present in Our Cohort

| ID | Nucleotide Change | Amino Acid Change | ACMG Classification | | | | | Allele Frequency (Genome) | | | | | Functional Prediction | | | | | | | | ClinVar ID | Comment |
| --- | --- | --- | --- | --- | --- | --- | --- | --- | --- | --- | --- | --- | --- | --- | --- | --- | --- | --- | --- | --- | --- | --- |
|  |  |  | Verdict | Criteria | | | |  |  |  |  |  | Revel | | | Mutation Taster | | | Conservation | SpliceAI |  |  |
|  |  |  |  | Factor 1 | Factor 2 | Factor 3 | Factor 4 | Allele No | African | Asian | European | Total | Prediction | Score | Rankscor | Prediction | Score | Converted | PhyloP100way |  |  |  |
| 20 | c.10873C>G | p.Leu3625Val | VUS*^a^* | PM2 | PP4 |  |  | 22 | 0 | 0.000686 | 0 | 0.0000885 | Benign | 0.439 | 0.7431 | Uncertain | 0.9999 | Disease causing | 2.021 | Benign 0 | 505145 | ×3 VUS |
| 8 | c.11241_11242insG | p.Val3747fs* | Likely path | PVS1 | PM2 |  |  | 0 | 0 | 0 | 0 | Absent | NA |  |  | NA |  |  | 1.356 | Splice-Altering (0.23) | NO |  |
| 23 | c.1239-1G>T | NA | Pathogenic | PVS1 | PM2 | PP5 |  | 2 | 0 | 0 | 0.0000294 | 0.0000131 | NA |  |  | Uncertain | 1 | Disease causing | 9.703 | Splice-Altering (0.89) | 1067661 | ×1 likely path |
| 24 | c.12706G-T | p.Glu4236* | Likely path | PVS1 | PM2 |  |  | Absent | Absent | Absent | Absent | Absent | NA |  |  | Uncertain | 1 | Disease causing | 2.745 | Benign (0) | NO |  |
| 11 | c.16111delA | p.Ser5371fs* | Likely path | PVS1 | PM2 |  |  | Absent | Absent | Absent | Absent | Absent | NA |  |  | NA |  |  | 4.85 | Benign (0) | NO |  |
| 24 | c.16387dup | p.Tyr5463Leufs*8 | Likely path | PVS1 | PM2 |  |  | Absent | Absent | Absent | Absent | Absent | NA |  |  | NA |  |  | 1.923 | Benign (0) | NO |  |
| 4 | c.16453_16454del | p.Gln5485Aspfs*2 | Likely path | PVS1 | PM2 |  |  | Absent | Absent | Absent | Absent | Absent | NA |  |  | NA |  |  | 4.648 | Benign (0) | NO |  |
| 11 | c.8156-1460A>G | NA | VUS | PM2 | PM3 | PP4 |  | Absent | Absent | Absent | Absent | Absent | NA |  |  | NA |  |  | 1.406 | Splice-Altering (0.72) | NO |  |
| 10 | c.2070G>A | p.Trp690* | Likely path | PVS1 | PM2 |  |  | Absent | Absent | Absent | Absent | Absent | NA |  |  | Deleterious | 1 | Disease causing | 9.757 | Benign (0) | NO |  |
| 4 | c.2239A>G | p.Arg747Gly | VUS | PM2 | PP3 | PP4 |  | 1 | Absent | Absent | 0.00000887 | 0.00000402 | Benign | 0.36 | 0.6805 | Uncertain | 1 | Disease causing | 8.996 | Splice-Altering (0.65) | 1382993 | ×1 VUS |
| 16 | c.2758C>T | p.Arg920* | Pathogenic | PVS1 | PM2 | PP5 |  | 1 | Absent | Absent | 0.0000147 | 0.00000658 | NA |  |  | Uncertain | 1 | Disease causing | 3.192 | Benign (0.01) | 1454895 | ×1 path |
| 22 | c.2849del | p.Gly950Glufs*30 | Likely path | PVS1 | PM2 |  |  | Absent | Absent | Absent | Absent | Absent | NA |  |  | NA |  |  | 3.629 | Benign (0) | NO |  |
| 8 | c.3290-1G>A | NA | Likely path | PVS1 | PM2 |  |  | Absent | Absent | Absent | Absent | Absent | NA |  |  | Uncertain | 1 | Disease causing | 9.712 | Splice-Altering (0.99) | NO |  |
| 25 | c.3460T>A | p.Trp1154Arg | Likely path | PM2 | PM3 | PP3 | PP4 | Absent | Absent | Absent | Absent | Absent | Pathogenic | 0.729 | 0.9 | Uncertain | 1 | Disease causing | 7.791 | Benign (0.07) | NO |  |
| 15 | c.3726_27insA | p.Phe1242fs* | Pathogenic | PVS1 | PM2 | PP5 |  | Absent | Absent | Absent | Absent | Absent | NA |  |  | NA |  |  | 3.116 | Benign (0.01) | 1454044 | ×1 path |
| 1 | c.4553_4554del | p.Gln1518fs* | Likely path | PVS1 | PM2 |  |  | Absent | Absent | Absent | Absent | Absent | NA |  |  | NA |  |  | 9.055 | Benign (0) | NO |  |
| 13 | c.6458_6466delins8 | p.Lys2153Argfs*7 | Likely path | PVS1 | PM2 |  |  | Absent | Absent | Absent | Absent | Absent | NA |  |  | NA |  |  | NA | NA | NO |  |
| 12 | c.6466delG | p.Ala2156Leufs*4 | Likely path | PVS1 | PM2 |  |  | Absent | Absent | Absent | Absent | Absent | NA |  |  | NA |  |  | 7.955 | Benign (0.08) | NO |  |
| 20 | c.7087G>A | p.Glu2363Lys | VUS*^a^* | PM2 | PP4 |  |  | Absent | Absent | Absent | Absent | Absent | Uncertain | 0.49 | 0.7772 | Uncertain | 1 | Disease causing | 9.722 | Uncertain (0.12) | NO |  |
| 16 | c.9749-2delA | NA | Pathogenic | PVS1 | PM2 | PP5 |  | Absent | Absent | Absent | Absent | Absent | NA |  |  | NA |  |  | 7.715 | Splice-Altering (0.94) | 1098772 | ×3 path/likely path |
| 28 | c.10750delC | p.His3584Ilefs*11 | Likely path | PVS1 | PM2 |  |  | Absent | Absent | Absent | Absent | Absent | NA |  |  | NA |  |  | 5.376 | Benign (0.01) | NO |  |
| 28 | c.11808_11809insG | p.Leu3937Alafs*32 | Likely path | PVS1 | PM2 |  |  | Absent | Absent | Absent | Absent | Absent | NA |  |  | NA |  |  | 3.373 | Benign (0.01) | NO |  |
| 27 | c.13433G>T | p.Ser4478Ile | VUS | PM2 | PP3 | PP4 |  | 1 | 0.0000241 | Absent | Absent | 0.00000657 | Bening | 0.262 | 0.5748 | Uncertain | 1 | Disease causing | 9.661 | Splice-Altering (0.8) | NO |  |
| 3 | c.11563G>T | p.Glu3855* | Likely path | PVS1 | PM2 |  |  | Absent | Absent | Absent | Absent | Absent | NA |  |  | Uncertain | 1 | Disease causing | 0.355 | Benign (0.01) | NO |  |
| 17 | c.8347G>T | p.Glu2783* | Pathogenic | PVS1 | PM2 | PP5 |  | Absent | Absent | Absent | Absent | Absent | NA |  |  | Uncertain | 1 | Disease causing | 7.72 | Benign (0.05) | 1712024 | ×1 likely path |
| 6 | c.14004_14007delCTTT | p.Phe4668Leufs*18 | Likely path | PVS1 | PM2 |  |  | Absent | Absent | Absent | Absent | Absent | NA |  |  | NA |  |  | 5.753 | NA | NO |  |

ACMG = American College of Medical Genetics and Genomics, NA = not available/applicable, path = pathogenic, VUS = variant of uncertain significance.

*^a^*In *cis* with loss-of-function pathogenic variant.
